# Kelulut Honey-Incorporated Hybrid Gelatin-PVA Hydrogel for Wound Healing: Fabrication and In Vitro Characterization

**DOI:** 10.3390/polym17192618

**Published:** 2025-09-27

**Authors:** Andik Nisa Zahra Zainuddin, Raniya Razif, Aifa Asyhira Khairul Nizam, Manira Maarof, Nur Izzah Md Fadilah, Yang-Hee Kim, Ebrahim Mahmoudi, Mh Busra Fauzi

**Affiliations:** 1 Department of Tissue Engineering and Regenerative Medicine, Faculty of Medicine, Universiti Kebangsaan Malaysia, Cheras 56000, Kuala Lumpur, Malaysia; p153345@siswa.ukm.edu.my (A.N.Z.Z.); raniyarazif@gmail.com (R.R.); asyhira@gmail.com (A.A.K.N.); manira@ukm.edu.my (M.M.); izzahfadilah@ukm.edu.my (N.I.M.F.); 2 Advance Bioactive Materials-Cells UKM Research Group, Universiti Kebangsaan Malaysia, Bangi 43600, Selangor, Malaysia; 3 Bone and Joint Research Group, Centre for Human Development, Stem Cells and Regeneration, Institute of Developmental Sciences, University of Southampton, Southampton SO16 6YD, UK; yanghee.kim@soton.ac.uk; 4 Department of Chemical and Process Engineering, Faculty of Engineering and Built Environment, Universiti Kebangsaan Malaysia, Bangi 43600, Selangor, Malaysia; ebi.dream@gmail.com

**Keywords:** hydrogel, Kelulut honey, in vitro, Kelulut honey-wound healing mechanisms, Kelulut honey-physicochemical properties

## Abstract

Hydrogels are attractive biomaterials for skin replacement and tissue regeneration, offering advantages over split-skin grafts for large or irregular wounds. Honey-containing hydrogels are of particular interest, combining honey’s natural healing properties with the versatility of hydrogel matrices. This study aimed to develop a biocompatible, biodegradable, and mechanically stable hydrogel as a cutaneous substitute. To achieve this, different formulations were prepared using gelatin (GE), polyvinyl alcohol (PVA), and Kelulut honey (KH). The formulations were designated as: GE-PVA (6% (*w*/*v*) GE: 5% (*w*/*v*) PVA, without KH), GE-PVA-H1 (containing 1% (*v*/*v*) KH), GE-PVA-H5 (containing 5% (*v*/*v*) KH), and GE-PVA-H10 (containing 10% (*v*/*v*) KH). All formulations were crosslinked with 0.1% (*w*/*v*) genipin (GNP). GE-PVA-H1 and GE-PVA-H1-GNP showed swelling ratios of 110.18 ± 20.14% and 86.31 ± 14.27%, lower than GE-PVA-H5 (125.79 ± 23.76%), GE-PVA-H10 (132.79 ± 20.86%), and their crosslinked counterparts. All formulations had WVTR <1500 g/m^−2^h^−1^, with GE-PVA-H1-GNP at 501.21 ± 41.35 g/m^−2^h^−1^, GE-PVA-H5-GNP at 473.77 ± 44.10 g/m^−2^h^−1^, and GE-PVA-H10-GNP at 467.51 ± 73.59 g/m^−2^h^−1^. GE-PVA-H1-GNP exhibited the slowest biodegradation (0.0036 ± 0.0003 g/h vs. 0.0096–0.0206 g/h for other groups). Contact angle was lowest for GE-PVA-H1-GNP (38.46° ± 3.89°), confirming higher hydrophilicity compared with GE-PVA-H5/H10 groups. Resilience (98.85% ± 1.03%) and compression strength (77.42% ± 7.17%) of GE-PVA-H1-GNP were comparable to GE-PVA-H5-GNP and GE-PVA-H10-GNP. MTT assays confirmed cytocompatibility across all groups. Collectively, GE-PVA-H1-GNP emerged as the optimal formulation, combining mechanical stability, hydrophilicity, and biocompatibility for wound healing applications.

## 1. Introduction

The skin is a sophisticated and multifunctional organ that protects the body from external threats while maintaining physiological stability [1]. It also regulates temperature, perceives pain stimuli, and preserves moisture for homeostasis [2]. However, prolonged exposure to external elements can strain this barrier, increasing susceptibility to damage. When compromised, as in wounds, the skin may contribute to various illnesses [3]. A wound is an injury that results in tissue loss or damage, which can impact internal organs as well as muscles. Depending on the healing process and duration, a wound can be classed as either acute or chronic [4,5]. Acute wounds result from cuts, tears, or abrasions, while chronic wounds often stem from underlying conditions such as diabetes, poor circulation, or immune deficiencies [6,7]. These chronic injuries are expensive and necessitate long-term treatment; the wound care market is expected to grow at a 6.6% annual pace from 2020 to 2027, reaching $18.7 billion [8]. Coagulation, inflammation, proliferation, and maturation are the normal steps in the healing process; however, chronic wounds frequently stay in the inflammatory stage, which prevents tissue regeneration [9]. High reproducible performance, superior biocompatibility, robust cell adhesion, and appropriate mechanical qualities are all necessary for an ideal wound treatment.

Effective care is essential for both acute and chronic wounds, involving not only direct treatment but also management of comorbidities and prevention of complications that may arise from the injury [10]. Choosing the right dressing is crucial for faster healing, lower costs, and improved quality of life. Gauze and bandages are examples of passive, traditional wound dressings that work well on clean, dry wounds [11]. However, they often foster bacterial growth, delay healing, increase expenses, and contribute to antibiotic resistance due to limited drainage [12]. To address these drawbacks, modern dressings were developed with semi-permeability and highly absorbent layers. These advanced materials enhance granulation tissue formation and support epithelial cell migration from wound edges, thereby accelerating closure [13]. They also maintain a stable temperature and humidity, mimicking the body’s natural environment, protect fragile granulation tissue, promoting cell growth and differentiation, and limit scarring. In addition, modern dressings act as a barrier against external bacteria, effectively reducing the risk of cross-infection [5].

Considering these characteristics, hydrogels are well suited for modern wound dressings [14]. These 3D networks of hydrophilic polymers maintain a moist environment that supports granulation and re-epithelialization in tissue regeneration [15]. They offer strong adhesion, shape adaptability, and mechanical protection for effective wound coverage, yet can be removed and replaced with minimal trauma to the wound bed. Hydrogels also promote gas exchange, enhancing cellular activity, and their high-water retention, tissue-like structure, and drug delivery capacity extend their applications to tissue engineering and other biomedical fields [3,14,16]. Hydrogels can be derived from natural or synthetic polymers. Natural types are biocompatible and biodegradable but often lack mechanical strength and may trigger immune responses [17]. Man-made polymers including polyvinyl alcohol (PVA), polyethylene glycol (PEG), polyethylene oxide (PEO), and polyacrylamide (PAAM) are used to make synthetic hydrogels. These hydrogels often offer better stability and strength, and some, like PAAM, also offer biocompatibility [18]. On the other hand, hybrid hydrogels combine natural and synthetic components to overcome individual limitations. By mimicking the extracellular matrix, they provide biocompatibility, biodegradability, mechanical integrity, and gelation under physiological conditions. Their controlled swelling, suitable porosity, and ability to deliver bioactive molecules make them particularly promising for wound healing and tissue engineering [19].

Gelatin, derived from the partial hydrolysis of collagen, is obtained from animal sources such as bones, cartilage, tendons, ligaments, and skin of cattle, pigs, fish, or chickens. As a natural polymer containing the RGD (Arg-Gly-Asp) sequence, it promotes cell adhesion and proliferation [20,21]. As a natural mimic of the extracellular matrix (ECM), gelatin-based hydrogels aid wound healing by absorbing red blood cells, triggering clotting, providing antimicrobial effects, and enabling sustained release of growth factors like bFGF [22,23]. However, gelatin’s rapid degradation and hydrophilic surface limit its effectiveness as a wound dressing, so it is often combined with synthetic polymers such as polyvinyl alcohol (PVA) to create more stable and durable hydrogels [24]. PVA is a synthetic, water-soluble long-chain polymer derived from poly (vinyl acetate). PVA hydrogels form 3D crosslinked networks with low toxicity, high water absorption, strong mechanical strength, and good biocompatibility, making them useful for biomedical applications [25]. Genipin, a natural crosslinker, improves the stability and mechanical strength of biopolymers like chitosan and gelatin while reducing degradation, making them more suitable for food packaging, wound dressings, and tissue engineering [26].

Honey has long been used in treating burns, ulcers, abscesses, and chronic wounds, mainly due to its antibacterial, immunomodulatory, and tissue-repairing effects [27]. It contains water, sugars, proteins, wax, pollen, and minerals, combining bioactive components that collectively support healing [28]. Honey comes in two types, depending on the bee species: one made by stinging bees (Apis) and the other by stingless bees (*Trigona*). *Kelulut* honey, produced by stingless bees of the *Trigona* genus within the Meliponini tribe, is widely recognized in Malaysia [29,30]. Compared to Apis honey, it has higher moisture content, typically ranging between 25 and 35% versus 17–20% in honeybee honey, along with a slightly sour taste and stronger therapeutic potential. Packed with phenolic chemicals, especially polyphenols like flavonoids and phenolic acids, it counteracts free radicals at wound sites, preserves cellular structures, and encourages cell growth. Compared to Tualang and Gelam honey, this high antioxidant activity ranks slightly lower the Manuka [31,32,33,34]. In addition, Kelulut honey exhibits broad antibacterial activity, linked to hydrogen peroxide, low pH, and high osmolarity, with potency comparable to Manuka honey through both peroxide and non-peroxide mechanisms [31,35]. It also contains anti-inflammatory compounds, including phenolics like kaempferol and caffeic acid, which help reduce inflammatory responses and support tissue repair [36].

While previous research has explored honey-containing hydrogels, most studies have focused on Manuka or other traditional honeys, often combined with additional bioactive agents for wound healing applications [37,38]. However, there is limited research on hydrogels incorporating Kelulut honey, despite its distinct chemical composition and therapeutic potential. Therefore, this study aimed to fill this gap by developing Kelulut honey-containing hydrogels, with or without genipin crosslinking, and systematically evaluating their physicochemical and biological properties for wound healing applications. The hydrogels were formulated using gelatin and PVA with genipin as a crosslinker, and the impact of varying Kelulut honey concentrations was examined. This approach sought to create a simple, accessible, and reproducible hydrogel system while identifying a formulation with suitable performance and biocompatibility for skin tissue engineering, as assessed through in vitro testing.

## 2. Materials and Methods

The study design was approved by the Universiti Kebangsaan Malaysia Research Ethics Committee (Code no. JEP-2024-904). 

### 2.1. Preparation of Gelatin-PVA-Kelulut Honey Hydrogel

To achieve a 5% (*w*/*v*) concentration, 0.5 g of PVA powder (MERCK KGaA, Darmstadt, Germany; partially hydrolysed [≥85%], MW 70,000 g/mol) was dissolved in 10 mL of distilled water (dH_2_O) for one hour at 60 °C. After cooling to 40 °C, 6 g of bovine gelatin (GE) powder from Nitta-Gelatin Ltd. in Osaka, Japan, was weighed and added to the dissolved PVA mixture. It was then agitated for 30 min until it was completely dissolved. The dissolved Gelatin-PVA mixture was then mixed with 0.1 mL of Kelulut honey (Kuching, Sarawak, Malaysia) to achieve a 1% (*v*/*v*) concentration. To get concentrations of 5% (*v*/*v*) and 10% (*v*/*v*), respectively, the procedure was repeated for 0.5 mL and 1 mL of Kelulut honey. Next, 0.01 g (*w*/*v*) of genipin (GNP) powder was dissolved in 70% ethanol (EtOH; MERCK, Darmstadt, Germany) to create 0.1% of GNP (FUJIFILM Wako Pure Chemical Corporation, Chuo-Ku, Osaka, Japan). The final formulation of GE-PVA-GNP, GE-PVA-H1-GNP (1% Kelulut Honey), GE-PVA-H5-GNP (5% Kelulut Honey), and GE-PVA-H10-GNP (10% Kelulut Honey) was obtained by adding GNP to the Gelatin-PVA-Honey mixture once it had become homogenous. In contrast, the non-crosslinked hydrogels (without GNP) were represented as GE-PVA, GE-PVA-H1 (1% Kelulut Honey), GE-PVA-H5 (5% Kelulut Honey), and GE-PVA-H10 (10% Kelulut Honey). The same standardised procedure was used to prepare each hydrogel formulation, and the same batches and suppliers of reagents were used to guarantee uniformity and repeatability from batch to batch.

### 2.2. Gross Appearance Evaluation

Both crosslinked and non-crosslinked formulations, with and without Kelulut honey (KH), were visually evaluated in order to determine how KH affected the hydrogel’s final look. The KH-loaded hydrogels (GE-PVA-H1-GNP, GE-PVA-H1, GE-PVA-H5-GNP, GE-PVA-H5, GE-PVA-H10-GNP, and GE-PVA-H10) were compared to the control hydrogels (GE-PVA and GE-PVA-GNP) in terms of their gross morphology. Digital cameras were used to take cross-sectional and top-view pictures (Nikon, Tokyo, Japan).

### 2.3. Evaluation of Gelation Time

To optimise the process, the gelation time of every hydrogel group was measured. Following the dissolution of PVA, gelatin, and Kelulut honey, 2 mL of the non-crosslinked hydrogel solution was pipetted into glass vials. The solution was monitored as its temperature was allowed to reach 27 °C, at which point a timer was initiated. The solution was observed continuously until a gel-like structure formed, after which the timer was stopped, and the time and temperature at which gelation occurred were recorded. Gelation was confirmed by inverting the glass vials; the absence of any flow of the hydrogel solution indicated complete gelation. The same procedure was applied to the crosslinked groups, with GNP added to the solution and allowed to fully dissolve before testing.

### 2.4. Swelling Ratio

In order to evaluate the hydrogel’s capacity to absorb wound exudates, which is a critical component in wound healing applications, its swelling behavior was examined. The swelling behavior of the hydrogels was examined using a technique modified from an earlier work in order to evaluate their ability to absorb fluids [39]. First, the initial weight (Wi) of the wet hydrogels was determined by weighing them. After that, they spent 24 h at room temperature immersed in Dulbecco’s phosphate-buffered saline (DPBS; pH = 7.4). The hydrogels were then weighed once again to ascertain their final weight (Wf) after the extra DPBS was carefully removed using filter paper (Whatman^®^, No. 42, Merck, Darmstadt, Germany). The following formula was used to determine the swelling ratio percentage:

Swelling Ratio(%)=((Wf−Wi))/Wi×100

### 2.5. Water Vapor Transmission Rate (WVTR)

The hydrogels were put through a Water Vapour Transmission Rate (WVTR) test in accordance with the American Society for Testing and Materials (ASTM) standard to assess their capacity to transfer water and promote gas exchange, two processes that are critical for wound healing [40]. At first, the glass cylinder opening’s area was expressed in square meters. The hydrogel was then placed over the aperture in cylinder (A) after 10 mL of distilled water (dH_2_O) had been supplied. The initial weight of the complete structure was then noted when the hydrogel was parafilm-sealed onto the aperture (Wi). After that, the sealed cylinders were kept for 24 h at 37 °C in an incubator with a 5% CO_2_ controlled environment. The cylinders were weighed once more to ascertain the ultimate weight following this incubation period, enabling the computation of the WVTR (Wf). The following formula was used to determine the WVTR:

WVTR=((Wi−Wf)/A)×t

### 2.6. Enzymatic Biodegradation

The hydrogels’ biodegradability after being exposed to an enzyme reaction was assessed using enzymatic biodegradation testing. A technique modified from an earlier work was used to examine the hydrogels’ biodegradation [41]. The test used dry scaffolds that had been cut in half, and each half-hydrogel (1/2) had been freeze-dried for three days beforehand. A 0.6% collagenase stock solution (Worthington, Lakewood, NJ, USA) was used to create a 0.0006% (*w*/*v*) collagenase type-I solution. After recording the dry scaffolds’ initial weight (Wi) for every hydrogel group, the scaffolds were put into a 16-well plate. Two millilitres of the 0.0006% collagenase solution were added to each dry scaffold, completely submerging it. For six hours, the plate was kept at room temperature. To get rid of any remaining collagenase, the scaffolds were cleaned with DPBS following this incubation time. After being pre-frozen for two hours at −80 °C, the scaffolds were freeze-dried for twelve to twenty-four hours. To determine the degree of biodegradation, the final weight of the freeze-dried scaffolds was noted (Wf). The following formula was then used to get the biodegradation rate:

Biodegradation Rate:(Wi−Wf)/t

### 2.7. Contact Angle

Hydrogels in their wet form were used to determine the contact angle. Each hydrogel was positioned with its top surface on a level surface and its bottom (smooth) surface exposed and facing up. The hydrogel’s exposed surface was gently covered with a 10 μL drop of distilled water. As soon as the water droplet made contact with the hydrogel surface, an image of it was taken. The contact angle of this image was then calculated using ImageJ software (version 1.54k, NIH, Bethesda, MD, USA), which provided information on the hydrophilicity of the hydrogel surface.

### 2.8. Porosity

As explained below, porosity study was carried out on freeze-dried hydrogels made using two different preparation techniques.

#### 2.8.1. SEM

Samples were initially freeze-dried and then lightly coated with gold to enhance imaging quality in order to study the interior structure of the hydrogels. The hydrogels were next scanned using a high-resolution field-emission scanning electron microscope (FESEM, Supra 55VP model, Jena, Germany) in accordance with a previously defined technique. The ImageJ software (version 1.5, Bethesda, MD, USA) was used to measure and analyse the pore sizes [42].

#### 2.8.2. Liquid Displacement

Before the hydrogels’ porosity was evaluated, they were lyophilised and evaluated using a liquid displacement method that was slightly modified from Ghaffari et al. [43]. Since absolute ethanol (99.5% EtOH) can pass through the hydrogel pores without causing the matrix to shrink or swell, it was chosen as the displacement medium. The freeze-dried hydrogels’ initial weight (Wi) and volume (V) were measured prior to a 24 h immersion in pure ethanol. The post-immersion weight (Wf) was noted after the surplus ethanol was carefully removed using filter paper (Whatman^®^, No. 42, Merck, Darmstadt, Germany) following incubation. After that, porosity was determined using the given formula:

$$Porosity\left(\%\right)=\left(\frac{Wf-Wi}{\rho V}\right)\times 100$$

In the equation, ρ is the density of ethanol (0.789 g/cm^3^), V is the scaffold’s volume, Wf is the scaffold’s final weight after ethanol immersion, and Wi is its beginning dry weight.

### 2.9. Mechanical Testing (Compression and Resilience)

Compression and resilience testing of the hydrogels was conducted using a method adapted and modified from a previously published protocol [44]. The compression test involved placing the hydrogel on a flat surface and applying a consistent weight of 300 g to the sample. After the hydrogels were fabricated in their wet form and removed from the silicone molds, they were placed on a flat surface. A photograph was taken to document the scaffold’s condition “before compression” (Ai). A 300 g weight was then applied to the scaffold for 1 min. During this time, when the weight was on the top of hydrogel, another photograph was taken to capture the hydrogel “after compression” (Ac). Afterward, the weight was removed, and a final photograph was taken to capture the scaffold “after resilience” (Af). The area of the scaffold at each stage was measured using ImageJ software to analyze the effects of compression and hydration. The compression and resilience of the scaffold were calculated using the following formula:

Compression ratio(%)=(Ac/Ai)×100

Resilience ratio(%)=(Af/Ai)×100

### 2.10. Sample Characterization

A Fourier Transform Infrared (FTIR) spectrometer (PE, Waltham, MD, USA) was used to identify the hydrogels’ functional groups within the wavelength range of 4000 cm^−1^ to 500 cm^−1^. The chemical composition and any post-crosslinking alterations were ascertained by analysing the absorbance peaks. The composition of elements of the hydrogels was also evaluated using energy dispersive X-ray (EDX) analysis. A Phenom Pro X SEM EDX microscope (Phenom, Eindhoven, The Netherlands) was used for this analysis. The control samples were commercial gelatin, PVA powder, Kelulut honey, and Genipin (GNP).

### 2.11. Thermostability Analysis

The Shimadzu TGA-50 (model TGA-50, Shimadzu, Kyoto, Japan) equipment was used to perform thermogravimetric analysis (TGA). The dynamic tests were conducted with a temperature range of 25 °C to 200 °C in a nitrogen atmosphere. To evaluate the hydrogels’ thermal stability, they were heated steadily at a rate of 10 °C per minute. The sample weight loss as a function of temperature was recorded through continuous monitoring. TA60W (v7.0) software was used to analyse the resultant data.

### 2.12. Skin Cell Isolation and Culture

As excess tissue after abdominoplasty surgeries, skin samples were taken from four consented patients. Dulbecco’s phosphate-buffered saline (DPBS) was used to sterilise the skin after it was sliced into tiny pieces (1–2 cm). After the tissue was digested for 4–6 h using 0.6% collagenase Type I (Worthington-Biochemical Corporation, 730 Vassar Ave Lakewood, NJ, USA) in a shaker incubator, it was treated for 10 min with trypsin-EDTA (Gibco, USA). A Dulbecco’s Modified Eagle’s Medium/Nutrient Mixture F-12 (DMEM/F12) supplemented with 10% foetal bovine serum (FBS; Gibco/BRL, Carlsbad, CA, USA) was used to resuspend the pellet that was made after the cell suspension was centrifuged for five minutes at 5000 rpm. After being seeded at a density of 1 × 10^4^ cells/cm^2^ into a 6-well culture plate, the cells were incubated at 37 °C with 5% CO_2_. Every two to three days, the medium was changed. The cells were grown, trypsinised, and differentiated in a 75 cm^2^ culture flask containing F12: DMEM and 10% FBS when they had attained 70–80% confluency.

### 2.13. Preparation of Sterile Gelatin-PVA-Kelulut Honey Hydrogel

Hydrogels were made in a biosafety cabinet to guarantee sterility. Sterile gelatin (GE) from Nitta-Gelatin Ltd. in Osaka, Japan, polyvinyl alcohol (PVA) from MERCK KGaA in Germany (partially hydrolysed (≥85%), MW 70,000 g/mol), Kelulut honey, and genipin (GNP) powder from Fujifilm Wako Pure Chemical Corporation in Japan were all used in the procedure. First, a 5% (*w*/*v*) concentration solution was made by dissolving 0.5 g of sterile polyvinyl alcohol (PVA) powder in 10 mL of sterile distilled water (dH_2_O) at 60 °C. A 6% (*w*/*v*) gelatin-PVA (GPVA) stock solution was then created by adding 0.6 g of sterile gelatin (GE) powder to the PVA solution at 40 °C and stirring until it was completely dissolved and homogenous. The gelatin-PVA solution was then mixed with 0.1 mL of sterile Kelulut honey to achieve a 1% (*v*/*v*) concentration. Repeating this procedure with 0.5 mL of Kelulut honey allowed for a 5% (*v*/*v*) concentration. 0.01 g of sterile genipin (GNP) (FUJIFILM Wako Pure Chemical Corporation, Chuo-Ku, Osaka, Japan) was dissolved in 70% ethanol (EtOH; MERCK, Darmstadt, Germany) to crosslink the hydrogels. The homogenous gelatin-polyvinyl alcohol-Kelulut honey (GE-PVA-H) mixture was then mixed with this genipin solution. Third, GE-PVA-GNP, GE-PVA-H1-GNP (1% Kelulut honey), and GE-PVA-H5-GNP (5% Kelulut honey) were the final formulations of the sterile crosslinked hydrogels.

### 2.14. Cytotoxicity Test

The 3-(4,5-dimethylthiazol-2-yl)-2,5-diphenyl tetrazolium bromide (MTT) assay (Merck, Darmstadt, Germany) was used to evaluate the impact of Kelulut honey on cell viability. The assay evaluated the response of human dermal fibroblasts (HDFs) to three crosslinked hydrogel formulations: GE-PVA-GNP, GE-PVA-H1-GNP (1% Kelulut honey), and GE-PVA-H5-GNP (5% Kelulut honey). HDFs were cultivated at 37 °C with 5% CO_2_ after being seeded onto sterile hydrogels at a density of 1.5 × 10^6^ cells. On days 1, 5, and 7, 200 μL of fresh medium containing 20 μL of 0.5 mg/mL MTT solution (Sigma-Aldrich, St. Louis, MO, USA) was added in place of the culture medium. To dissolve the formazan crystals, 100 μL of dimethyl sulfoxide (DMSO; Sigma-Aldrich, St. Louis, MO, USA) was added to the medium after the samples had been incubated for 4 h at 37 °C. To assess cell viability and proliferation, the plates were shaken for ten minutes at 37 °C. An ELISA microplate reader was used to quantify the absorbance at 540 nm.

### 2.15. Proliferation of Fibroblasts

In accordance with the manufacturer’s instructions, the encapsulated cells within the hydrogels were assessed for cell viability and proliferation using a Live/Dead Cytotoxicity Assay for mammalian cells (Thermo Fisher Scientific, Waltham, MA, USA). Primary human dermal fibroblasts (HDFs) of passage 3 were seeded into the hydrogels at a cell density of 1.5 ×10^6^ cells per millilitre. 24 h after incubation, cell viability was evaluated at 37 °C in a controlled setting with 5% CO_2_. Cell toxicity was assessed using a fluorescence microscope (Nikon A1R-A1, Tokyo, Japan) at ×20 magnification following 40 min of treatment with 200 μL of a mixed working solution containing 1.5 μL acetoxymethoxy calcein derivative (calcein-AM) and 6 μL ethidium homodimer-1 (EthD-1) diluted with 2992.5 μL of pure medium at 37 °C.

### 2.16. Statistical Analysis

GraphPad Prism software (version 10.0, GraphPad Software Inc., San Diego, CA, USA) was used to do statistical analysis. Significant group differences were assessed using both one-way and two-way ANOVA. The statistical significance threshold is set at *p* < 0.05, and quantitative data are presented as mean ± standard deviation (SD). Every experiment was carried out with independent replicates in at least three copies (N = 3).

## 3. Results

### 3.1. Gross Morphology (Top and Side View) and Gelation Time

**Figure 1:**
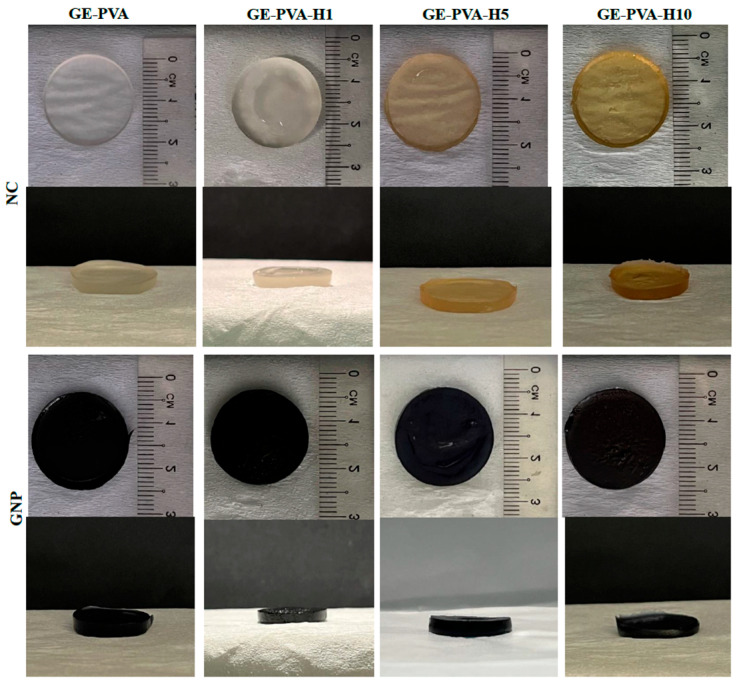
A gross morphology of the top and side views of the genipin-crosslinked (GNP) and non-crosslinked (NC) hydrogels for the GE-PVA, GE-PVA-H1, GE-PVA-H5, and GE-PVA-H10 formulations.

From top and side viewpoints, Figure 1 shows the gross morphology of all hydrogel formulations, including crosslinked and non-crosslinked varieties, with and without Kelulut honey (KH). While non crosslinked formulations exhibit a clear to yellowish hue that intensifies with increasing KH concentration, crosslinked hydrogels have a light blue to teal colouring. GE-PVA hydrogels without KH, in both crosslinked and non-crosslinked forms, were used as controls. The visual assessment confirms that all hydrogel samples possess a semi-solid texture, rather than a fluid-like consistency. The hydrogels from each group were also firm and gelatinized perfectly after being peeled from the silicone mold. GE-PVA-H10 exhibits a distinct sticky structure and the most pronounced yellowish hue, resulting from its highest KH concentration among the non-crosslinked groups.

### 3.2. Gelation Time

**Figure 2:**
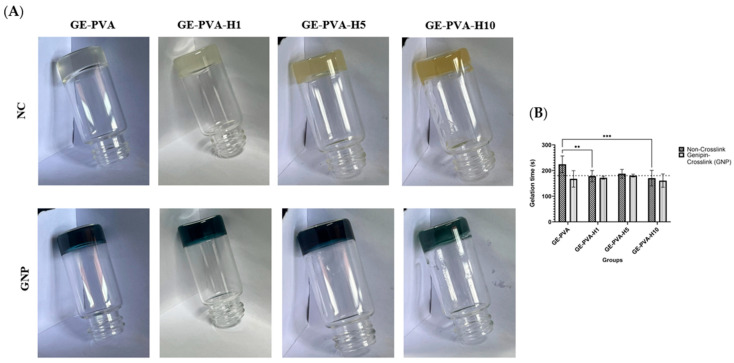
Gelation time analysis of GE-PVA, GE-PVA-GNP, GE-PVA-H1, GE-PVA-H1-GNP, GE-PVA-H5, GE-PVA-H5-GNP, GE-PVA-H10, and GE-PVA-H10-GNP. (**A**) The inverted jar method to analyze the time of gelation of non-crosslinked (NC) and genipin-crosslinked (GNP) hydrogels for GE-PVA, GE-PVA-H1, GE-PVA-H5, and GE-PVA-H10 formulations. (**B**) Time of gelation of non-crosslinked (NC) and genipin-crosslinked (GNP) hydrogels for GE-PVA, GE-PVA-H1, GE-PVA-H5, and GE-PVA-H10 formulations (** indicates *p* = 0.0033 and *** indicates *p* = 0.0005).

The results of the inverted tube experiment are shown in Figure 2A, which also shows the gelation behaviour of GE-PVA formulations with different concentrations of Kelulut honey (KH). For crosslinked samples, 0.1% genipin was added. The images show the hydrogels after a fixed inversion period, offering a qualitative evaluation of gel formation.

Moreover, Figure 2B illustrates the gelation times of various hydrogel formulations, comparing non-crosslinked (NC) and genipin-crosslinked (GNP) groups, both with and without Kelulut honey (KH). All GNP-crosslinked hydrogels demonstrated faster gelation than their non-crosslinked counterparts, highlighting the accelerating effect of 0.1% genipin. Among all formulations, GE-PVA-H10-GNP exhibited the fastest gelation time (160.63 ± 25.97 s), followed by GE-PVA-H1-GNP (172 ± 5.70 s), both well below the 180 s threshold. While all KH-containing formulations achieved gelation within this critical limit, GE-PVA-H1 (178 ± 22.06 s) and GE-PVA-H5-GNP (180.5 ± 5.26 s) were the most optimal, approaching the 180 s mark. These results suggest that both genipin crosslinking and higher KH concentrations synergistically enhance gelation efficiency.

### 3.3. Physicochemical Analysis

**Figure 3:**
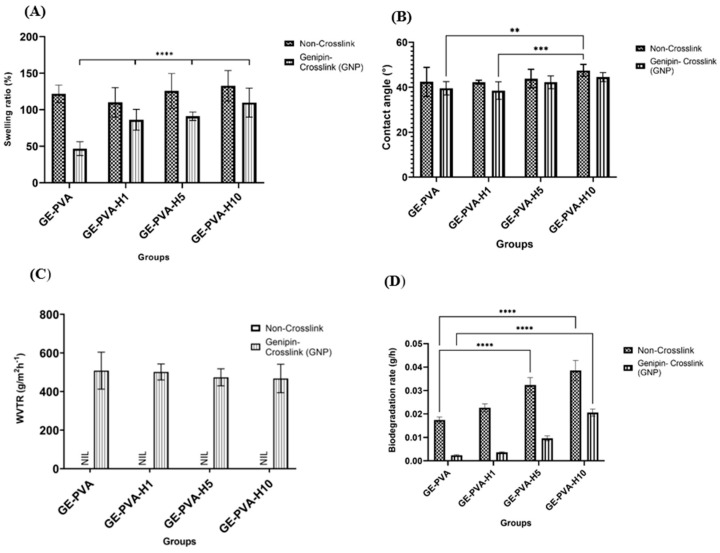
Physicochemical analysis of GE-PVA, GE-PVA-GNP, GE-PVA-H1, GE-PVA-H1-GNP, GE-PVA-H5, GE-PVA-H5-GNP, GE-PVA-H10, and GE-PVA-H10-GNP. (**A**) Percentage of swelling ratio. (**B**) Contact angle (°). (**C**) Water vapor transmission rate (WVTR) (g/m^−2^h^−1^). (**D**) Biodegradation rate (g/h). ** indicates *p* = 0.0023, *** indicates *p* = 0.0004 and 0.0008, and **** indicates *p* < 0.0001.

The swelling ratio, contact angle, water vapour transmission rate (WVTR), and biodegradation rate of gelatin–PVA–Kelulut honey (KH) hydrogels, comprising crosslinked and non-crosslinked formulations, were evaluated to determine their physicochemical properties. The quantitative outcomes of KH-incorporated hydrogels and those without KH are comprehensively compared in Figure 3.

The hydrogel’s capacity to absorb liquids, including Dulbecco’s Phosphate-Buffered Saline (DPBS), is indicated by the swelling ratio, which also shows the crosslinking density and liquid retention capacity. As shown in Figure 3A, all non-crosslinked hydrogels exhibited higher swelling ratios than their genipin-crosslinked counterparts. Among these, GE-PVA-H10 (non-crosslinked) showed the highest swelling capacity (132.79 ± 20.86%), while GE-PVA-H10-GNP recorded the highest value within the crosslinked group (109.83 ± 19.84%). The most pronounced reduction due to crosslinking was observed in GE-PVA-GNP, confirming that crosslinking strongly influences water uptake. In comparison, GE-PVA-H1-GNP and GE-PVA-H5-GNP displayed swelling ratios of 86.31 ± 14.27% and 91.19 ± 5.72%, respectively. The crosslinked formulations showed highly significant differences (*** *p* < 0.0001) according to statistical analysis, with GE-PVA-H10-GNP showing a significantly greater swelling ratio than the others. These findings suggest that greater Kelulut honey incorporation substantially enhances water absorption, even after crosslinking.

Figure 3B presents the contact angle measurements for various GE–PVA–Kelulut honey (KH) hydrogels, encompassing both crosslinked and non-crosslinked formulations. All of the samples showed contact angles that were less than 90°, which suggests that the surface was hydrophilic. GE-PVA-H10 had the highest value of contact angle among the non-crosslinked formulations (47.45° ± 2.64°), while GE-PVA-H10-GNP had the highest among the crosslinked group (44.52° ± 2.01°). An increasing trend in contact angle was observed with higher KH concentrations, suggesting a gradual reduction in surface hydrophilicity. For instance, GE-PVA-H1 and GE-PVA-H1-GNP recorded contact angles of 42.19° ± 0.83° and 38.46° ± 3.89°, respectively, whereas GE-PVA-H5 and GE-PVA-H5-GNP measured 43.80° ± 4.14° and 42.17° ± 2.86°, respectively. The addition of Kelulut honey (KH), particularly at higher concentrations, increased the hydrogel’s hydrophobic qualities by further increasing the contact angle. Significantly. GE-PVA-H10 had a much greater contact angle than GE-PVA-H1-GNP, as shown by a highly significant difference (*** *p* = 0.0008), indicating that adding 10% KH considerably decreases hydrophilicity by raising the surface contact angle. Additionally, a significant difference (*** *p* = 0.0004) between GE-PVA-GNP and GE-PVA-H10 was noted, reinforcing the effect of increased KH concentrations on the wettability of the hydrogel surface.

The water vapor transmission rate (WVTR) test evaluates the permeability of the hydrogel dressing to water vapor and gases. As illustrated in Figure 3C, among the formulations, the control group without Kelulut honey (GE-PVA-GNP) demonstrated the highest WVTR value at 508.11 ± 96.02 g/m^−2^h^−1^. For the KH-incorporated hydrogels, GE-PVA-H1-GNP exhibited the highest WVTR (501.21 ± 41.35 g/m^−2^h^−1^), followed by GE-PVA-H5-GNP (473.77 ± 44.10 g/m^−2^h^−1^) and GE-PVA-H10-GNP (467.51 ± 73.59 g/m^−2^h^−1^). Although a decreasing trend in WVTR was observed with increasing KH concentration, suggesting reduced permeability, there are no statistically significant differences found among the groups.

Gelatin–PVA–based hydrogels, including crosslinked and non-crosslinked formulations with and without Kelulut honey (KH), have biodegradation rates that are depicted in Figure 3D. Overall, non-crosslinked samples demonstrated faster degradation rates compared to their genipin (GNP)-crosslinked counterparts. The control hydrogels, GE-PVA and GE-PVA-GNP, exhibited the lowest degradation rates at 0.0174 ± 0.0013 g/h and 0.0024 ± 0.0002 g/h, respectively. In contrast, the incorporation of KH increased the degradation rate in both crosslinked and non-crosslinked groups. For example, GE-PVA-H1 degraded at 0.0227 ± 0.0017 g/h, while its crosslinked counterpart, GE-PVA-H1-GNP, degraded at 0.0036 ± 0.0003 g/h. Significant variations between the several formulations were found by statistical analysis. In the non-crosslinked group, GE-PVA-H5 exhibited a significantly higher degradation rate than GE-PVA (****, *p* < 0.0001), suggesting that 5% KH notably enhanced biodegradation. Similarly, a significant difference was observed between GE-PVA and GE-PVA-H10 (****, *p* < 0.0001), where the higher KH concentration further accelerated degradation. Among crosslinked hydrogels, GE-PVA-H10-GNP showed a significantly faster degradation rate than GE-PVA-GNP (****, *p* < 0.0001), indicating that KH can still effectively enhance biodegradability even in the presence of GNP crosslinking. These findings collectively suggest that increasing concentrations of Kelulut honey contribute to elevated biodegradation rates, with the effect persisting in both crosslinked and non-crosslinked hydrogel systems.

### 3.4. Mechanical Strength of the Fabricated Hydrogels

**Figure 4:**
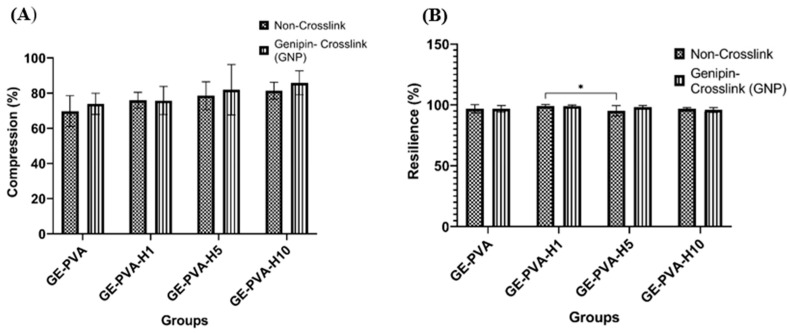
Mechanical strength analysis of GE-PVA, GE-PVA-GNP, GE-PVA-H1, GE-PVA-H1-GNP, GE-PVA-H5, GE-PVA-H5-GNP, GE-PVA-H10, and GE-PVA-H10-GNP. (**A**) Compression percentage. (**B**) Percentage of resilience. * indicates *p* = 0.0369.

To assess the hydrogels’ mechanical strength, a crucial factor for possible implantation applications, compression testing was performed, and the findings are shown in Figure 4A. No statistically significant changes in compressive strength between the different scaffold formulations were found by the analysis. All hydrogels were capable of withstanding mechanical loading, with non-crosslinked variants supporting approximately 69% to 81%, and crosslinked variants withstanding around 73% to 85% of the applied 300 g load, for both with and without Kelulut honey (KH), indicating consistent mechanical integrity across all groups.

The hydrogels’ resilience, assessed through a single-cycle compression–recovery test, reflects their capacity to regain their original structure after deformation, as demonstrated in Figure 4B. Statistical analysis identified a single significant difference (*, *p* = 0.0369) in resilience between GE-PVA-H1 and GE-PVA-H5, which exhibited 99.04% ± 1.22 and 95.13% ± 4.20% recovery, respectively. Despite this, all hydrogel formulations demonstrated excellent resilience, with complete or near-complete shape recovery observed across the groups.

### 3.5. Chemical Characterization

Fourier-transform infrared (FTIR) spectroscopy provides characteristic molecular fingerprints for identifying polymers, crosslinkers, and their corresponding chemical bonds. The method is based on the interaction of the sample with infrared radiation through absorption and transmission. The spectra of these gelatin-based hydrogels show distinct amide and hydroxyl bands, indicating their structural makeup.

**Figure 5:**
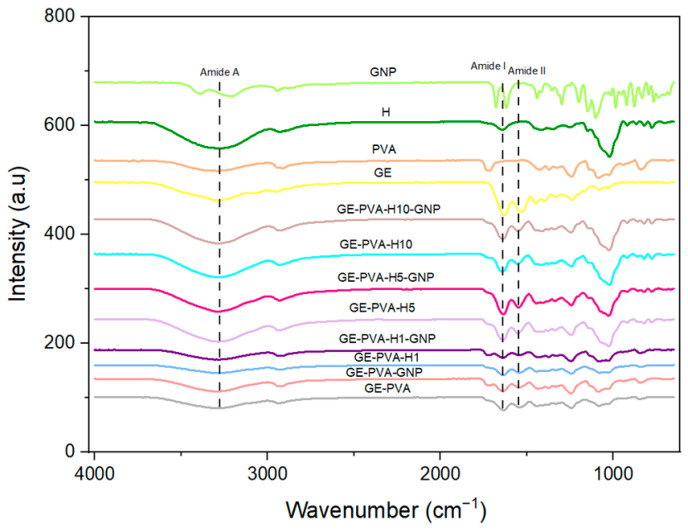
FTIR spectra illustrates the chemical characterization of the fabricated hydrogels.

Based on Figure 5, in both genipin-crosslinked and non-crosslinked samples, with or without Kelulut honey, hydrogen bonding within the hydrogel matrix is reflected by the broad absorption band seen at 3280–3500 cm^−1^, which correlates to O–H and N–H stretching (Amide A). Gelatin’s protein backbone is confirmed by the distinctive Amide I band (1625–1650 cm^−1^, C=O stretching) and Amide II band (1525–1545 cm^−1^, N–H bending and C–N stretching). All hydrogel groups consistently exhibit a clear peak at 1545 cm^−1^, which denotes leftover gelatin. Furthermore, the scaffold’s structural integrity is further supported by C–O stretching bands (1080–1030 cm^−1^) and C–H bending vibrations (1400–1450 cm^−1^).

Moreover, displaced peaks at 1054 cm^−1^ and 1650 cm^−1^, which correspond to C–O stretching and C=O stretching, respectively, are seen in all samples containing Kelulut honey, both crosslinked and non-crosslinked. Additional interactions associated with sugar are suggested by the emergence of peaks within 875–699 cm^−1^ that are attributed to C–O and C–H vibrations. Furthermore, all PVA-containing samples’ FTIR spectra show peaks linked to C–H stretching at 2918.97 cm^−1^ and 1729.83 cm^−1^.

### 3.6. 3D-Microporous Structure Hydrogel

**Figure 6:**
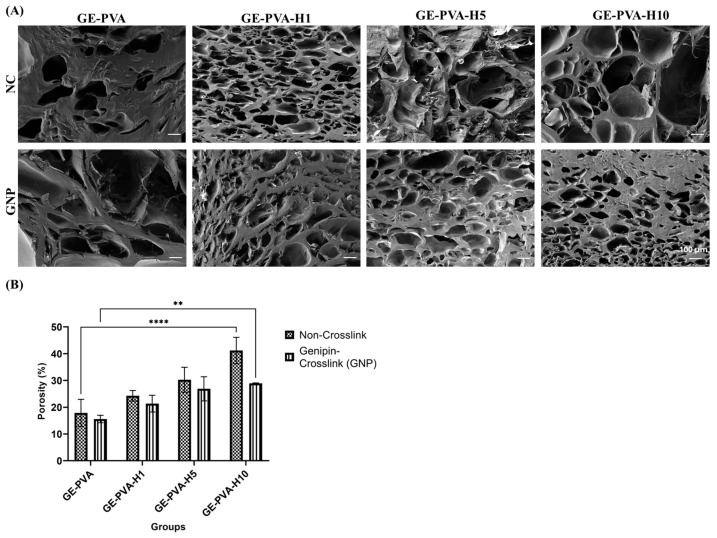
3D-microporous structure of GE-PVA, GE-PVA-GNP, GE-PVA-H1, GE-PVA-H1-GNP, GE-PVA-H5, GE-PVA-H5-GNP, GE-PVA-H10, and GE-PVA-H10-GNP. (**A**) SEM micrographs of the hydrogels at 100× magnification, illustrating their cross-sectional microporous structure. (**B**) Porosity percentage. ** indicates *p* = 0.0066, and **** indicates *p* < 0.0001.

Heterogeneous porosity architectures were shown by SEM micrographs of gelatin-PVA-based hydrogels with and without genipin crosslinking and Kelulut honey (KH) (Figure 6A). In non-crosslinked groups, GE-PVA showed large, irregular pores, while KH addition led to smaller, more uniform pores in GE-PVA-H1 and larger, disrupted pores in GE-PVA-H10. Crosslinked samples exhibited better-defined pores and greater surface roughness, in which, GE-PVA-H1-GNP formed a balanced, interconnected network compared to GE-PVA-GNP, whereas higher KH concentrations (GE-PVA-H5-GNP, GE-PVA-H10-GNP) produced rougher, highly porous structures due to matrix disruption. Overall, crosslinked hydrogels demonstrated superior pore definition and interconnectivity compared to non-crosslinked counterparts.

Overall porosity was raised in a concentration-dependent manner by adding Kelulut honey (KH), in both genipin-crosslinked and non-crosslinked hydrogels (Figure 6B). Across all formulations, non-crosslinked hydrogels consistently exhibited higher porosity than crosslinked counterparts. Statistical analysis confirmed these trends, with GE-PVA-H10 showing significantly greater porosity than GE-PVA (****, *p* < 0.0001) in the non-crosslinked group, and GE-PVA-H10-GNP displaying significantly higher porosity than GE-PVA-GNP (**, *p* = 0.0066) among crosslinked samples, indicating that higher KH concentrations enhance porosity regardless of crosslinking.

The elemental makeup of gelatin–PVA hydrogels, both genipin-crosslinked and non-crosslinked, with and without Kelulut honey was determined by EDX analysis (Table 1). The carbon content was significantly reduced by the addition of Kelulut honey. Among the non-crosslinked samples, GE-PVA-H10 showed the lowest carbon level (52.65 ± 0.21), while GE-PVA-H10-GNP exhibited the lowest value among the genipin-crosslinked hydrogels (52.40 ± 0.42). In contrast, oxygen content increased progressively with higher honey concentrations. The highest oxygen levels were observed in GE-PVA-H10 (47.35 ± 0.21) for the non-crosslinked group and in GE-PVA-H10-GNP (47.60 ± 0.42) for the crosslinked group. Nitrogen content displayed a decreasing trend with increasing honey concentration. GE-PVA-H5 recorded the lowest nitrogen level among the non-crosslinked hydrogels (1.50 ± 1.13), while GE-PVA-H1-GNP showed the lowest value among the crosslinked group (3.90 ± 0.14). Notably, nitrogen was undetectable in GE-PVA-H5-GNP, GE-PVA-H10, and GE-PVA-H10-GNP. These variations confirm successful honey incorporation and indicate that elemental composition is influenced by both honey concentration and crosslinking state.

### 3.7. Thermogravimetric (TGA) Analysis

**Figure 7:**
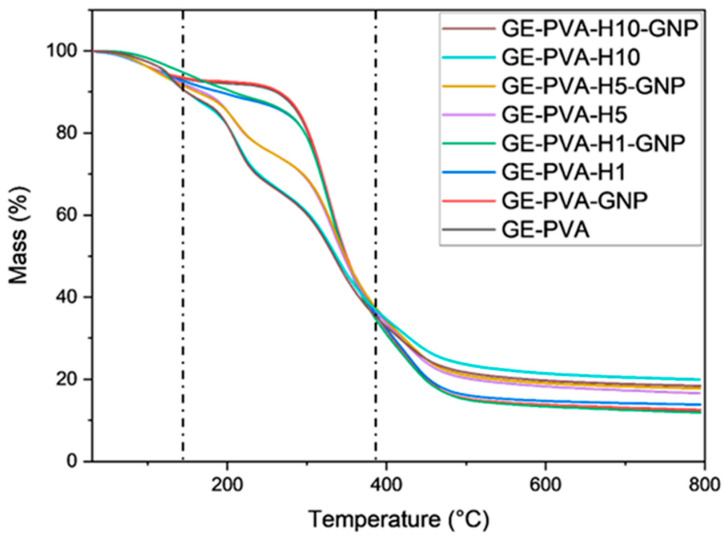
TGA study of the various hydrogel groups demonstrating their degradation behaviour and thermal stability. The dotted line indicates the three degradation phases: (1) moisture evaporation (30–150 °C), (2) polymer backbone decomposition (200–380 °C), and (3) further degradation (400–490 °C).

The thermal stability and degradation behaviour of GE-PVA-based hydrogels, both with and without genipin crosslinking and Kelulut honey, were assessed by thermogravimetric analysis (TGA). As shown in Figure 7, the first phase, corresponding to moisture evaporation, occurred between 30 and 150 °C. GE–PVA and GE–PVA–GNP exhibited mass losses of 3.02% and 3.01%, respectively. In comparison, the honey-containing formulations showed slightly lower values, with GE–PVA–H1–GNP (1.22%), GE–PVA–H1 (2.23%), GE–PVA–H5–GNP (2.90%), GE–PVA–H5 (2.96%), GE–PVA–H10–GNP (1.99%), and GE–PVA–H10 (2.05%). The lowest initial mass loss was observed for GE–PVA–H1–GNP (1.22%). During the second degradation phase, corresponding to the main polymer backbone decomposition, all GE–PVA-based hydrogels exhibited major degradation peaks within a narrow range of 200–380 °C. Weight losses for GE–PVA–GNP and GE–PVA were 34.62% and 35.21%, respectively. The honey-containing formulations showed higher mass losses, with GE–PVA–H1–GNP (34.40%), GE–PVA–H1 (35.55%), GE–PVA–H5–GNP (39.51%), GE–PVA–H5 (40.43%), GE–PVA–H10–GNP (47.14%), and GE–PVA–H10 (47.94%). In all pairs, GNP-crosslinked samples exhibited slightly lower mass losses than their non-crosslinked counterparts. In the third degradation phase, occurring between 400 and 490 °C, further weight losses were recorded. The residual mass at 600 °C for GE–PVA–GNP and GE–PVA was 86.20% and 86.53%, respectively. The honey-containing formulations showed residual masses of GE–PVA–H1–GNP (86.64%), GE–PVA–H1 (85.31%), GE–PVA–H5–GNP (80.97%), GE–PVA–H5 (81.75%), GE–PVA–H10–GNP (79.99%), and GE–PVA–H10 (80.03%).

### 3.8. Cytotoxicity and Proliferation of Fibroblasts

**Figure 8:**
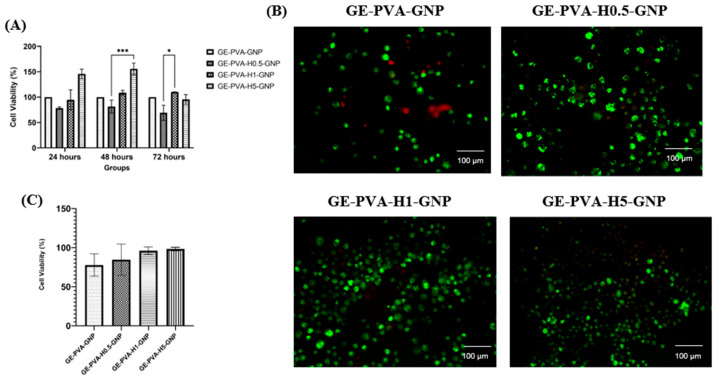
Cytotoxicity and proliferation of fibroblasts on GE-PVA, GE-PVA-GNP, GE-PVA-H1, GE-PVA-H1-GNP, GE-PVA-H5, GE-PVA-H5-GNP, GE-PVA-H10, and GE-PVA-H10-GNP. (**A**) MTT assay results showing the cytotoxicity of GE-PVA-genipin (GNP)-based hydrogels supplemented with varying concentrations of Kelulut honey (0.5%, 1%, and 5% *v*/*v*) toward human dermal fibroblasts. FDC represents the control medium. Statistical differences are denoted as *** *p* = 0.0002 and * *p* = 0.0321. (**B**) Live/dead assay results showing the proliferation of HDFs through green and red fluorescence staining. (**C**) HDFs’ cell viability using a live/dead assay.

The viability of fibroblasts cultured on GE-PVA-genipin (GNP) hydrogels incorporated with different concentrations of Kelulut honey (0.5%, 1%, and 5% *v*/*v*) was evaluated using two methods: the MTT assay at 24, 48, and 72 h and the live/dead assay at 24 h (Figure 8A, Figure 8B and Figure 8C, respectively). For MTT assay (Figure 8A), at 24 h, GE-PVA-H5-GNP exhibited the highest cell viability (145.8 ± 9.48%), exceeding that of the control (GE-PVA-GNP, 100%), while GE-PVA-H0.5-GNP showed the lowest viability (78.3 ± 2.37%). At 48 h, both GE-PVA-H1-GNP (108.9 ± 4.50%) and GE-PVA-H5-GNP (155.7 ± 11.43%) demonstrated higher viability compared to the control, with GE-PVA-H5-GNP showing a statistically significant increase than GE-PVA-H0.5-GNP (*** *p* = 0.0002). In contrast, GE-PVA-H0.5-GNP remained the lowest (81.5 ± 12.83%). At 72 h, GE-PVA-H1-GNP maintained the highest cell viability (110.2 ± 0.24%), while GE-PVA-H5-GNP dropped to 95.5 ± 9.53%, comparable to the control. GE-PVA-H0.5-GNP displayed the lowest viability (69.2 ± 14.91%) and was significantly different from GE-PVA-H1-GNP (* *p* = 0.0321). Importantly, all formulations showed cell viability above the ISO.

The live/dead experiment (Figure 8B,C) showed no signs of cytotoxicity for any of the hydrogels that were evaluated after a 24 h incubation period. Viable cells were visualized by green fluorescence, while red fluorescence denoted non-viable cells, confirming the overall biocompatibility of the formulations. Among the groups, GE-PVA-H5-GNP exhibited the greatest cell viability (98.22 ± 2.17%), followed by GE-PVA-H1-GNP (96.07 ± 4.74%), GE-PVA-H0.5-GNP (84.46 ± 20.03%), and GE-PVA-GNP (77.62 ± 14.28%).

## 4. Discussion

Tissue engineering has appeared as a novel regenerative strategy in modern medicine, offering new possibilities for restoring and repairing damaged tissues. This approach involves the utilization of various biomaterials designed to support, guide, and enhance the growth and regeneration of functional tissues and organs [45]. Tissue-engineered skin substitutes (TESS), such as hydrogels, can replace damaged skin by replicating key structural and functional features, including flexibility, barrier protection, and regulation of transepidermal water loss. Designed to mimic the 3D extracellular matrix (ECM), they provide a microenvironment that supports cell proliferation and migration, thereby promoting wound healing [46,47]. The goal of this research is to create a hybrid gelatin-PVA hydrogel with Kelulut honey for wound healing and as a possible formulation for usage in injectable hydrogel and 3D-bioprinting applications in the future. This biomaterial’s novel idea combines the structural and mechanical stability of the gelatin-PVA network with the natural substance Kelulut honey, which is known for its capacity to promote tissue regeneration. It is anticipated that this dual function will hasten the healing process of wounds by promoting cell division, aiding in tissue restoration, and creating an environment that is conducive to regeneration. Furthermore, hybrid hydrogels that combine natural and synthetic polymers with bioactive agents optimize healing by maintaining a balance between biocompatibility, biodegradability, and mechanical strength [1].

By integrating natural and synthetic polymers, gelatin, and PVA with different amounts of Kelulut honey, this study effectively created hydrogels that could be used as a treatment for chronic skin wounds. Together with gelatin, PVA, and Kelulut honey, genipin was added as a crosslinker to the hydrogel to improve its structural integrity and encourage cell proliferation, which in turn aids in wound healing [48,49]. The best formulations, GE-PVA-H1 and GE-PVA-H5-GNP, gelled at room temperature in three minutes (180 s). This time frame was chosen to allow physicians enough time to apply hydrogel to the wound site without causing premature solidification, which can obstruct extrusion in future applications using 3D bioprinting [50]. Also, the inclusion of genipin enhanced gelation across all hydrogel formulations by forming inter-chain covalent bonds with gelatin’s amino groups and other polymers, strengthening the hydrogel structure. This crosslinking mechanism supports consistent gelation within three minutes and offers the advantage of faster gelation compared to other methods, even at higher concentrations [51,52].

The physicochemical properties of the hydrogels were evaluated to assess their performance. Maintaining a moist environment, controlling exudate, and reducing patient discomfort are crucial factors in managing wounds since injured tissue frequently experiences significant water loss [53]. Hydrogels serve this function effectively by maintaining a moist environment at the wound site, thus supporting tissue regeneration [54]. The fabricated gelatin-PVA–Kelulut honey hydrogels, both genipin-crosslinked and non-crosslinked, exhibited acceptable swelling ratios, demonstrating their ability to absorb excess wound exudate. Although earlier studies suggested that wound-healing hydrogels should ideally achieve swelling levels of around 500% to prevent fluid accumulation, none of the formulations in this study reached that value, particularly when Kelulut honey was incorporated into the GE-PVA and GE-PVA-GNP networks [55]. Nevertheless, Zekry et al. reported that the optimal swelling ratio for honey-incorporated PVA-based hydrogels lies within 80–100%, which is consistent with the values observed for GE-PVA-H1-GNP and GE-PVA-H5-GNP. These findings suggest that while Kelulut honey incorporation may limit maximum swelling, it still allows the hydrogel to achieve an optimal range that supports wound healing, indicating a favorable balance between fluid absorption and structural stability [38]. Furthermore, the swelling ratio was found to gradually increase with higher Kelulut honey concentrations in both crosslinked and non-crosslinked formulations. This behavior is in line with earlier research showing that higher honey content promoted swelling because it raised the viscosity of the polymer and created a more porous hydrogel structure that allowed for greater solvent uptake [56]. The hygroscopic nature of Kelulut honey, attributed to its high sugar content, further contributes to this effect by promoting water absorption and improving the hydrophilic properties of the hydrogel [57,58]. These sugars include a lot of hydroxyl (-OH) groups, which easily form hydrogen bonds with water molecules to increase the hydrogel’s total capacity for absorption water [59].

The significance of surface characteristics in tissue regeneration was further highlighted by water contact angle analysis, which showed that the hydrogel’s surface properties, which are primarily determined by its water solubility and hydrogen-bonding capacity that determine its hydrophilicity or hydrophobicity. These properties then impact wettability, surface energy, and the capacity to support cell adhesion [60,61]. Although increasing the Kelulut honey concentration slightly shifts the balance toward hydrophobicity, GE-PVA-H1 and GE-PVA-H1-GNP exhibited lower contact angles than the GE-PVA and GE-PVA-GNP controls, indicating enhanced surface hydrophilicity. These findings are consistent with Mukhopadhyay et al., who reported that higher honey concentrations decreased hydrogel surface hydrophilicity, as reflected by increased contact angle values. This effect may result from disruption of the free water balance at the hydrogel surface, where the hygroscopic nature of honey alone is insufficient to fully maintain hydrophilicity [57]. GE-PVA-H1 and GE-PVA-H1-GNP exhibit enhanced hydrophilicity, suggesting that this is the ideal concentration of Kelulut honey for boosting surface hydrophilicity. This effect is probably caused by the presence of several hydrophilic substances in Kelulut honey, such as aliphatic organic acids, short-chain fatty acids, flavonoids, phenolic acids, polyphenols, and sugars like trehalulose [59]. Furthermore, the polar functional groups in flavonoids and phenolic substances, as well as the hydroxyl groups in sugars, can easily create hydrogen bonds with water molecules at the hydrogel surface [62]. This interaction elevates the hydrogel’s surface energy, increasing its affinity for water and enhancing wettability, as reflected by the lower contact angles in GE-PVA-H1 and GE-PVA-H1-GNP. These results agree with the swelling ratio findings, which also indicate that GE-PVA-H1-GNP represents the optimal honey concentration for maximizing water absorption. Although higher Kelulut honey concentrations slightly increased the contact angle, all formulations remained below 90°, indicating overall hydrophilicity. Incorporation of Kelulut honey enhanced surface hydrophilicity compared to the controls, demonstrating its potential to modulate gelatin-based hydrogels for improved interaction with the aqueous wound environment.

In addition, hydrogels must exhibit an appropriate water vapor transmission rate (WVTR) to maintain optimal wound moisture, which is essential for healing. Excessively high WVTR can dehydrate the wound, while too low WVTR may lead to exudate accumulation, emphasizing the need for controlled water loss in effective wound management [63]. An ideal WVTR for a hydrogel to perform well as a skin substitute is typically less than 1500 g m^−2^ h^−1^, which avoids dehydration while preserving the moisture-rich environment required for tissue regeneration, cell migration, and proliferation [64]. As a result, all of the fabricated GE-PVA–Kelulut honey–Genipin hydrogels demonstrated exceptional WVTR, with values between 468 and 510 g m^−2^ h^−1^, which is thought to be desirable for accelerating wound healing. This result is consistent with earlier research by Sutar et al., which found that WVTR values for normal skin were 204 g m^−2^/24 h and for wounded skin, 279 g m^−2^/24 h [65].

Since the hydrogels’ quick degradation after implantation is one of their main drawbacks, it was crucial to evaluate the hydrogels’ in vitro degradation. The enzymatic breakdown profiles of gelatin-PVA hydrogels with and without Kelulut honey are shown in Figure 3D with respect to genipin-crosslinked and non-crosslinked formulations. In contrast to hydrogels containing Kelulut honey, the controls, GE-PVA and GE-PVA-GNP, demonstrated higher durability and slower rates of degradation, suggesting that the inclusion of honey speeds up biodegradation. Nevertheless, all formulations-maintained degradation rates within the acceptable range of 0.01–0.1 g/h [16]. Furthermore, the biodegradation rate increased progressively with higher Kelulut honey concentrations in both crosslinked and non-crosslinked hydrogels. This trend is consistent with the swelling and contact angle results, where higher honey concentrations enhanced water uptake and surface wettability, thereby creating a more hydrated microenvironment that facilitated faster hydrogel breakdown [57]. In addition to honey’s natural water solubility, the existence of low-molecular-weight saccharides in Kelulut honey that readily dissolve in water enhanced its degradation process [66,67]. Moreover, genipin-crosslinked hydrogels, both with and without Kelulut honey, exhibited slower biodegradation rates compared to their non-crosslinked counterparts. This can be attributed to the genipin-mediated crosslinking, which reinforces the hydrogel network and provides greater resistance to enzymatic degradation, thereby enhancing scaffold stability [42].

The fabricated gelatin-PVA–Kelulut honey hydrogels, both genipin-crosslinked and non-crosslinked, successfully mimicked the mechanical behavior of normal skin, making them suitable for skin applications. Compression testing showed that adding Kelulut honey strengthened the hydrogels’ strength in comparison to the weaker GE-PVA and GE-PVA-GNP controls. A higher compression value indicated better resistance to deformation under load, reflecting enhanced mechanical stability. This enhancement can be explained by the presence of sugars, phenolic compounds, and other hydrophilic constituents in Kelulut honey, which introduce additional hydrogen bonding interactions with the polymer chains. Similar trends were reported in gellan gum–VCO–honey hydrogels, where increases in stress values and Young’s modulus were attributed to enhanced hydrogen bonding within the matrix [68]. Furthermore, genipin crosslinking contributed to scaffold stability by reinforcing the polymeric network, thereby providing additional resistance against mechanical stress [69]. These findings suggest that Kelulut honey, together with genipin crosslinking, reinforces hydrogels’ structural integrity, making them more resilient under compressive forces and well-suited for wound-healing applications. Moreover, all fabricated hydrogels demonstrated excellent resilience, with values exceeding 90%, indicating that they were able to recover at least 90% of their total area after compression. This highlights the ability of the scaffolds to maintain their structural integrity under repeated mechanical stress. Resilience, which measures the hydrogel’s ability to regain its original shape following compression, is mostly dictated by the crosslinking density and chain mobility of the polymer network. Only slight changes were seen in GE-PVA-H5, indicating that the addition of Kelulut honey, even at greater concentrations, did not significantly change this elastic behaviour (Figure 4B). Among the formulations, GE-PVA-H1 and GE-PVA-H1-GNP exhibited the best performance, regaining up to 99% of their total area after compression, suggesting that 1% Kelulut honey provided the optimal concentration for maintaining elasticity.

FTIR spectroscopy was employed to investigate the molecular structure of the gelatin–PVA hydrogels, both genipin-crosslinked and non-crosslinked, with and without Kelulut honey (KH). Gelatin, PVA, and genipin’s distinctive functional groups were seen in the spectra, together with clear signals that suggested interactions with Kelulut honey (Figure 5). O–H and N–H stretching (Amide A) is shown by the broad absorption band between 3280 and 3500 cm^−1^, which reflects hydrogen bonding within the hydrogel matrix and enhances mechanical strength and network stability. Characteristic gelatin peaks were observed at 1525–1545 cm^−1^ (Amide II, N–H bending and C–N stretching), with additional bands at 1400–1450 cm^−1^ (C–H bending) and 1030–1080 cm^−1^ (C–O stretching), confirming the protein backbone structure [70]. The band at 1625–1650 cm^−1^, typically assigned to Amide I (C=O stretching) in gelatin, was also influenced by carbonyl groups from sugars such as fructose in KH, suggesting overlapping contributions that support honey integration into the hydrogel matrix. The O–H stretching band shifted to about 3289 cm^−1^ once KH was added, suggesting that honey and gelatin had stronger hydrogen bonds. Sugar-related interactions from the glucose, sucrose, and fructose contained in KH were responsible for additional peaks at 1054 cm^−1^ (C–O stretching) and within the 875–699 cm^−1^ region (C–O and C–H vibrations) [57,71]. Additionally, all hydrogels containing PVA showed distinctive bands at 2918.97 cm^−1^ (C–H stretching) and 1729.83 cm^−1^ (C=O stretching), which is in line with earlier research by Masri et al. and validates the successful incorporation of PVA [72]. Collectively, these findings demonstrate that KH was effectively integrated into the hydrogel network, where its sugar-derived interactions and enhanced hydrogen bonding contributed not only to greater compressive stability but also to the potential biological functionality of the hydrogels.

The composition of elements of gelatin–PVA hydrogels, both genipin-crosslinked and non-crosslinked, with and without Kelulut honey (KH), was evaluated using an EDX analysis. The GE–PVA and GE–PVA–GNP controls displayed similar elemental profiles, consisting of ~60–63% carbon, 35–36% oxygen, and 10–12% nitrogen, which reflect the gelatin-based network structure. Both crosslinked and non-crosslinked formulations showed a substantial decrease in carbon and nitrogen concentrations and a rise in oxygen content upon the addition of 1, 5, and 10% KH. While this carbon trend contrasts with Razif et al., who reported an increase in carbon content with rising KH concentration due to the carbon-rich nature of honey, the observed decrease in nitrogen is consistent with their findings. The decrease in nitrogen indicates that some of the nitrogenous gelatin components were essentially replaced by KH sugars that were integrated into the hydrogel matrix [73]. The difference in carbon content may arise from the distinct composition of our formulations, which combined gelatin, PVA, and KH with or without genipin crosslinking. In addition, strong hydrogen bonding interactions between PVA hydroxyl groups, gelatin, and honey sugars may have promoted a more homogeneous distribution of KH within the hydrogel matrix, thereby reducing the surface exposure of carbon-rich sugar moieties and favoring oxygenated groups (–OH, –C=O) [74]. Since EDX provides relative elemental composition at the surface, the preferential exposure of oxygenated groups (–OH, –C=O) could account for the reduced carbon and increased oxygen percentages detected, despite the higher absolute carbon content expected from honey incorporation. The greater incorporation of oxygen further supports the presence of hydroxyl-rich sugar molecules from KH within the hydrogel network. Meanwhile, the relatively smaller change in carbon content at higher KH concentrations may suggest a shift in how honey integrates into the polymeric matrix, potentially involving more complex hydrogen bonding interactions or even minor phase separation.

SEM micrographs of gelatin-PVA-based hydrogels, both with and without genipin crosslinking and Kelulut honey (KH), exhibited heterogeneous porous structures (Figure 6A). Porosity measurements using the liquid displacement method further quantified the hydrogel porosity. Comparing the microstructures, GE-PVA and GE-PVA-GNP hydrogels displayed a more compact architecture than KH-containing hydrogels, with fewer but larger pores, indicating a denser network likely resulting from stronger gelatin–PVA–genipin interactions. Notably, GE-PVA-GNP hydrogels exhibited larger and less uniform pores than GE-PVA hydrogels, suggesting that genipin crosslinking influences pore size and distribution [72]. Crosslinked samples, regardless of KH presence, exhibited better-defined pores and greater surface roughness. In comparison to non-crosslinked hydrogels, the addition of genipin (GNP) increased the number of smaller pores in the crosslinked polymer chains by creating covalent interactions within the hydrogel network. Erdag et al. study, which found that hydrogels with lower GNP concentrations had more small pores than those with greater GNP concentrations, is consistent with this observation [75]. Subsequently, regardless of genipin crosslinking, hydrogels containing KH demonstrated improved pore interconnectivity and higher porosity. The creation of smaller, highly linked pores may be facilitated by interactions between KH and the gelatin matrix, which may either add more crosslinking points or lessen network stiffness. Furthermore, the hydrogen-bonding environment of water molecules inside the gelatin–PVA matrix may be changed by the high sugar content of KH, especially maltose and fructose, resulting in a less dense network structure with finer pores [59]. Non-crosslinked GE-PVA-H10 hydrogels, on the other hand, showed bigger, irregular pores at higher KH concentrations, indicating impaired structural homogeneity. This conclusion is consistent with research by Shamloo et al., who found that a surplus of honey can inhibit the development of a homogeneous sponge-like structure. Among crosslinked samples, GE-PVA-H1-GNP formed a balanced, interconnected network, whereas higher KH concentrations (GE-PVA-H5-GNP and GE-PVA-H10-GNP) resulted in rougher, highly porous architectures, indicating that excessive KH can still disrupt the crosslinked matrix [67].

Findings from porosity measurements by the liquid displacement method (Figure 6B) were consistent with SEM imaging. In both crosslinked and non-crosslinked hydrogels, porosity gradually rose as KH concentrations rose, indicating a concentration-dependent impact. According to Pinthong et al., this improvement could be explained by modifications in system viscosity and the moderately acidic nature of KH, which have an impact on the size and stability of gas bubbles during hydrogel formation [76]. Furthermore, the organic acids present in KH may reduce the efficiency of genipin-mediated crosslinking by lowering pH, thereby influencing final pore characteristics [77]. Crucially, porous hydrogel networks are essential for tissue engineering because they promote the elimination of metabolic waste, nutrition delivery, and cell infiltration [78]. In this study, porosity values obtained from the liquid displacement method closely matched the structural features observed by SEM (Figure 6A), reinforcing that KH incorporation effectively enhances porosity and interconnectivity, although high concentrations can compromise uniformity, particularly in non-crosslinked systems.

The thermal stability of GE–PVA-based hydrogels, with and without Kelulut honey and with or without genipin crosslinking, was assessed using thermogravimetric analysis (TGA). All formulations exhibited a three-step degradation profile (Figure 7). The first phase, between 30 and 150 °C, corresponded to moisture evaporation and was observed in all hydrogel samples. This initial mass loss is primarily due to water evaporation and may also involve the decomposition of thermally sensitive components in Kelulut honey, such as aromatic compounds [79]. During the second degradation stage, the hydrogels containing Kelulut honey exhibited greater weight loss than the GE–PVA and GE–PVA–GNP controls, indicating enhanced polymer backbone decomposition. This behavior can be attributed to the thermal instability of honey’s constituents. Simple sugars such as maltose, fructose, and glucose, which are abundant in Kelulut honey degrade at relatively low temperatures (~110–150 °C), producing volatile compounds like 5-hydroxymethylfurfural (HMF) [80]. In support of this, Koosha et al. stated that three different endothermic transitions were found at 168 °C, 232 °C, and 258 °C in DSC analysis of chitosan/PVA-honey films. Maltose, fructose, and glucose crystal melting was attributed to the 168 °C peak; PVA crystal melting was associated with the 232 °C transition, which was shifted upward by hydrogen bonding with honey; and honey-derived components like enzymes, minerals, or complex carbohydrate molecules were linked to the 258 °C peak [66]. These observations reinforce the interpretation that the presence of Kelulut honey accelerates polymer degradation, explaining the greater weight loss observed in TGA for honey-containing hydrogels. In the third degradation phase, GE–PVA–H10–GNP and GE–PVA–H10 exhibited the lowest weight losses among the crosslinked and non-crosslinked hydrogels, respectively, resulting in the highest residual masses at 600 °C. This outcome suggests that the higher honey content, particularly its sugar composition, contributes to improved thermal stability of the GE–PVA hydrogel network [68].

Cell viability evaluation represents a fundamental parameter in cytotoxicity testing, offering valuable insights into the biological responses of cells when exposed to potential toxicants or novel biomaterials. The incorporation of Kelulut honey into GE–PVA–GNP hydrogels exhibited a clear dose-dependent effect on fibroblast viability. After 24 h, the formulation with the highest honey concentration (GE–PVA–H5–GNP) demonstrated greater cell viability compared to the control (GE–PVA–GNP). This enhanced proliferation may be attributed to the rich bioactive profile of Kelulut honey, particularly its sugars, phenolic compounds, and flavonoids, which provide antioxidant protection and nutritional support, thereby promoting cellular growth and accelerating wound healing [81]. At 48 h, both GE–PVA–H1–GNP and GE–PVA–H5–GNP demonstrated higher fibroblast viability compared to the control. Notably, the GE–PVA–H5–GNP formulation not only achieved the highest viability at 24 h but also sustained this superior effect at 48 h, suggesting that Kelulut honey can offer a prolonged proliferative advantage. This effect is likely mediated by the continuous availability of honey-derived antioxidants and nutrients, such as flavonoids, phenolic acids, and natural sugars, which support cell growth over time [82]. By 72 h, however, a distinct trend emerged, in which only GE–PVA–H1–GNP maintained the highest cell viability, while GE–PVA–H5–GNP showed a sharp decline to levels comparable with the control. This indicates that prolonged exposure to higher concentrations of Kelulut honey may exert cytotoxic effects. The observed cytotoxicity may be associated with oxidative stress, as excessive honey content has been reported to trigger overproduction of reactive oxygen species (ROS), disrupting cellular homeostasis and impairing viability [73]. In addition, earlier studies have shown that elevated honey concentrations, due to their high sugar content and strong osmotic potential, can induce hyperosmotic stress, which has been reported to reduce microbial or cellular survival [83]. Importantly, despite these dose-dependent differences, all Kelulut honey–containing hydrogel formulations maintained cell viability above the ISO 10993-5 cytotoxicity threshold of 70%, confirming their non-cytotoxic nature. According to this international standard, materials are considered biocompatible if they maintain at least 70% cell viability in vitro. Therefore, the findings confirm that the developed hydrogels fall within a safe range, underscoring their potential as skin tissue engineering scaffolds [84].

In addition to the MTT assay, a Live/Dead assay was performed to further assess the impact of incorporating Kelulut honey into GE–PVA–GNP hydrogels. The results demonstrated that cell viability on GE–PVA–H0.5–GNP, GE–PVA–H1–GNP, and GE–PVA–H5–GNP hydrogels was comparable to that of the GE–PVA–GNP control, indicating the absence of significant cytotoxic effects. Moreover, no signs of cell membrane damage were observed at these honey concentrations (0.5%, 1%, and 5% *v*/*v*), as illustrated in Figure 8B. These findings support the notion that honey-based hydrogel dressing promote cell proliferation and contributes to accelerated wound healing. Honey-based hydrogel dressings enhance water absorption, cell growth, and bacterial inhibition, speeding up wound healing [85]. A greater percentage of viable cells in GE PVA H5 GNP were found by quantitative analysis of the Live/Dead assay (Figure 8C), indicating that this formulation initially offers a more conducive environment for cell survival. This effect might be explained by honey’s regulated release of H_2_O_2_, which can alter a number of cellular behaviours, such as migration, metabolic activity, signalling, and proliferation, all crucial processes for successful wound healing. Crucially, this observation is consistent with the 24 h MTT results, which showed that GE-PVA-H5-GNP had the highest cell viability [86]. Nevertheless, the MTT assay also showed that extended exposure was insufficient to maintain the proliferative benefit of GE-PVA-H5-GNP. The osmolality of stingless bee honey (SBH) rises with honey concentration, which explains the fall in viability seen in MTT at later time points. Osmotic imbalance at increasing SBH concentrations results in fluid outflow from the cells to keep the external environment in balance, which ultimately lowers survival when the SBH concentration beyond the ideal threshold. This explanation aligns with previous studies that detailed the cytotoxic impact of high honey content [87]. On the other hand, the most optimal formulation was the GE-PVA-H1-GNP hydrogel. MTT results verified that GE-PVA-H1-GNP maintained the best cell viability even at 72 h, while Live/Dead analysis demonstrated a high viability of almost 96% at 24 h. This implies that while minimising the cytotoxic stress linked to greater concentrations, modest integration of Kelulut honey offers enough bioactive compounds to promote wound healing and proliferation of cells. When combined, these results demonstrate that GE-PVA-H1-GNP is the best formulation, achieving the ideal ratio of bioactivity to biocompatibility for possible wound healing applications.

The biological function of Kelulut honey in wound healing is supported by prior research, even though this study did not specifically investigate molecular mechanisms. For instance, Kelulut honey has been demonstrated to prevent excessive keratinocyte migration and the epithelial–mesenchymal transition brought on by TGF-β. Kelulut honey has the potential to be a pro-healing and anti-scar agent since it may help avoid aberrant wound closure and keloid formation by modifying these processes [88]. Furthermore, in rats with chronic systemic inflammation caused by lipopolysaccharide (LPS, a bacterial endotoxin), Kelulut honey has been shown to reduce serum levels of pro-inflammatory markers, such as C-reactive protein (CRP), tumour necrosis factor-α (TNF-α), interleukin (IL)-1β, IL-6, IL-8, and monocyte chemoattractant protein-1 (MCP-1). Nuclear factor kappa-B (NF-κB), p38 mitogen-activated protein kinase (p38 MAPK), and nuclear factor erythroid 2–related factor 2 (Nrf2) signalling pathways were all modulated to produce these results. Since excessive inflammation frequently delays tissue regeneration, this modulation of inflammatory mediators and signalling cascades is extremely crucial to wound healing [89]. In addition to these benefits, Kelulut honey has potent antibacterial properties against pathogens that cause wounds. It was more effective than Tualang and Acacia honey at inhibiting the proliferation of *Pseudomonas aeruginosa*, *Escherichia coli*, and *Proteus mirabilis* at relatively low concentrations. Due in major part to its low pH (2.37 ± 0.13) and active peroxide concentration (H_2_O_2_), this honey variety from Malaysia showed the broadest antibacterial action against both Gram-positive and Gram-negative bacteria. Interestingly, compared to Gram-positive *Staphylococcus aureus,* the peroxide activity in Kelulut honey was very effective against Gram-negative *E. coli* [90]. Together, these mechanistic revelations offer a biological explanation for the encouraging in vitro results seen in this investigation, highlighting the potential of hydrogels based on Kelulut honey as multipurpose wound-healing dressings.

## 5. Conclusions

The Gelatin-PVA-Kelulut honey hydrogel was successfully developed with a quick gelation period of three minutes at room temperature (22–24 °C), suggesting that it can be used for a variety of applications, from sophisticated 3D bioprinting bioinks to traditional injectable hydrogels. The inclusion of water-soluble PVA, Kelulut honey, and genipin improved the hydrogel’s physicochemical performance, mechanical strength, and biological properties, supporting its potential as an implantable acellular biomatrix for wound healing applications. The hydrogels displayed enhanced hydrophilicity, appropriate swelling behavior, and efficient water absorption, enabling them to create and maintain a moist wound environment suitable for exudate management, particularly in chronic wound care. In vitro evaluation confirmed their compatibility with human dermal fibroblasts (HDFs), suggesting potential for promoting cellular wound repair. Nonetheless, further in vivo wound healing studies, particularly in chronic wound animal models, are necessary to validate long-term biocompatibility, degradation behavior, and therapeutic efficacy under physiologically relevant conditions. The possibility of enzymatic degradation in vivo may influence hydrogel stability, requiring optimization of degradation rates. Furthermore, comprehensive antimicrobial testing against common wound pathogens was not included in the current study; this will be addressed in subsequent research to completely establish the anti-infective capabilities of hydrogels based on Kelulut honey. Overall, Gelatin–PVA–Kelulut honey hydrogels demonstrate strong promise as multifunctional, biocompatible implantable biomatrices. Addressing current limitations will be essential to fully realize their translational and clinical potential, including future applications in injectable hydrogels and 3D bioprinting.

## Figures and Tables

**Table 1:** Elemental composition of the hydrogels determined by EDX analysis. All samples contained varying proportions of oxygen, carbon, and nitrogen. ND = not detected.

Hydrogels	C (%)	O (%)	N (%)
GE-PVA	60.15 ± 0.64	35.10 ± 0.28	12.40 ± 4.34
GE-PVA-GNP	63.35 ± 1.91	36.35 ± 2.33	10.42 ± 4.34
GE-PVA-H1	60.10 ± 4.10	36.50 ± 0.00	5.90 ± 0.00
GE-PVA-H1-GNP	60.20 ± 2.83	37.35 ± 0.21	3.90 ± 0.14
GE-PVA-H5	55.45 ± 0.35	42.60 ± 0.85	1.50 ± 1.13
GE-PVA-H5-GNP	56.30 ± 0.71	43.70 ± 0.71	ND
GE-PVA-H10	52.65 ± 0.21	47.35 ± 0.21	ND
GE-PVA-H10-GNP	52.40 ± 0.42	47.60 ± 0.42	ND

## Data Availability

The authors confirm that the data supporting the findings of this study are available within the article. Further inquiries can be directed to the corresponding author.

## Abbreviations

The following abbreviations are used in this manuscript:

WVTR	Water Vapour Transmission Rate
FTIR	Fourier Transform Infrared Spectroscopy
SEM	Scanning Electron Microscopy
ROS	Reactive Oxygen Species
3D	Three-Dimensional
H_2_O_2_	Hydrogen Peroxide
DPBS	Dulbecco’s Phosphate-Buffered Saline
TGA	Thermogravimetric Analysis
EDX	Energy Dispersive X-ray
C=O	Carbonyl Group
C-H	Carbon–Hydrogen Bond
C-O	Carbon–Oxygen Bond
C-N	Carbon-Nitrogen Bond
N-H	Nitrogen–Hydrogen Bond
O-H	Oxygen-Hydrogen Bond
OH	Hydroxyl group
K-H	Kelulut Honey
SBH	Stingless Bee Honey
HMF	5-hydroxymethylfurfural
